# Preclinical PET imaging of HIP/PAP using 1'-^18^F-fluoroethyl-*β*-D-lactose

**DOI:** 10.18632/oncotarget.20654

**Published:** 2017-09-06

**Authors:** Shaobo Yao, Yaping Luo, Zhenzhong Zhang, Guilan Hu, Zhaohui Zhu, Fang Li

**Affiliations:** ^1^ Department of Nuclear Medicine, Peking Union Medical College Hospital, Chinese Academy of Medical Science & Peking Union Medical College, Beijing, 100730, China; ^2^ Beijing Key Laboratory of Molecular Targeted Diagnosis and Therapy in Nuclear Medicine, Beijing, 100730, China

**Keywords:** PET, ^18^F-FEL, HIP/PAP, automated synthesis

## Abstract

**Purpose:**

This study aims at preclinical evaluation of a recently reported lactose analogue, 1'-^18^F-fluoroethyl-*β*-D-lactose (^18^F-FEL), in binding to hepatocarcinoma-intestine-pancreas and pancreatitis-associated protein (HIP/PAP) *in vitro* and *in vivo*.

**Methods:**

In this study, a multifunctional module was employed for the automated synthesis of ^18^F-FEL. Additional radiochemical purity, biodistribution, *in vitro* and *in vivo* competition, metabolic stability and micro-PET studies were performed using T3M4 and SK-BR-3 xenografts. Expression of HIP/PAP in T3M4 and SK-BR-3 tumor sections and cell lines were tested with immunohistochemistry (IHC) and western blot analysis.

**Results:**

The synthesis of ^18^F-FEL was completed in 30 min, with a radiochemical yield of 20 ± 5% and specific activity of 14.2 ± 7.1 GBq/*μ*mol. ^18^F-FEL exhibited high HIP/PAP-binding affinity with a half maximal inhibitory concentration (IC50) of 22.0 ± 4.0 nM. ^18^F-FEL demonstrated high stability and specific tumor accumulation, which was reduced by approximately 80% in a PET competition assay by co-injection of *β*-D-lactose. High expression of HIP/PAP was detected in T3M4 tumors and cell line, but negative result was found for SK-BR-3 cell line.

**Conclusion:**

^18^F-FEL has a high binding property to HIP/PAP, high stability and excellent pharmacokinetics *in vivo* and therefore warrants further evaluation in a proof-of-concept study in humans.

## INTRODUCTION

Despite the new cases of cancer keeps on an increasing trend ever since 1975, death rates have been falling on average 1.5% each year over 2005-2014 [1]. One of the key factors that contribute to the decline is early tumor diagnosis and sensitive imaging of tumor locations. Receptors that are uniquely expressed during cancer invasion and metastasis represent promising targets for cancer diagnosis or therapy. HIP/PAP, also known as REG 3A, is a 16 kD secreted plasma protein. HIP/PAP belongs to the group of VII of a family of proteins that contain a C-type lectin like domain, which binds to carbohydrates, and it is also known as “lactose-binding protein” [2, 3]. The overexpression of HIP/PAP has been linked to many different diseases including hepatocellular carcinoma [4–6], cholangiocarcinoma [7], glucagon-producing enteropancreatic endocrine tumors [8], pancreatic ductal adenocarcinoma [9], colorectal cancer [10], gastric adenocarcinomas [11] and biliary malignancies [12]. Therefore, HIP/PAP is a promising target for the detection of diseases above.

HIP/PAP has a high affinity to *β*-D-lactose and its analogues [13]. Three lactose analogues tracers: *β*-D-galactopyranosyl-(1,4')-2'-deoxy-2'-[^18^F]fluoro-*β*-D-glucopyranoside (^18^F-FDL), ethyl-*β*-D-galactopyranosyl-(1,4')-2'-deoxy-2'-[^18^F]fluoro-*β*-D-glucopyranoside (Et-^18^F-FDL) and 1'-^18^F-fluoroethyl-*β*-D-lactose (^18^F-FEL) had been reported to target mouse HIP/PAP in preclinical experiments [14–16]. The binding affinity of Et-^18^F-FDL and ^18^F-FEL to HIP/PAP expressed in the peritumal pancreatic acinar cells had been tested in an orthotopic L3.6pl-GL^+^ pancreatic tumor model and the findings were corroborated further using autoradiography and IHC assays [3, 13, 16-18]. However, these studies have limitations, such as complex synthetic steps that are not suitable for large-scale production and neglect of some pancreatic tumor cells expressing HIP/PAP [3, 17, 18].

In this study, we had synthesized a novel triflate-FEL precursor, established an automated radiosynthesis method of ^18^F-FEL, and proved the binding ability of ^18^F-FEL to HIP/PAP both *in vitro* and *in vivo*.

## RESULTS

### Radiosynthesis

**Figure 1:**
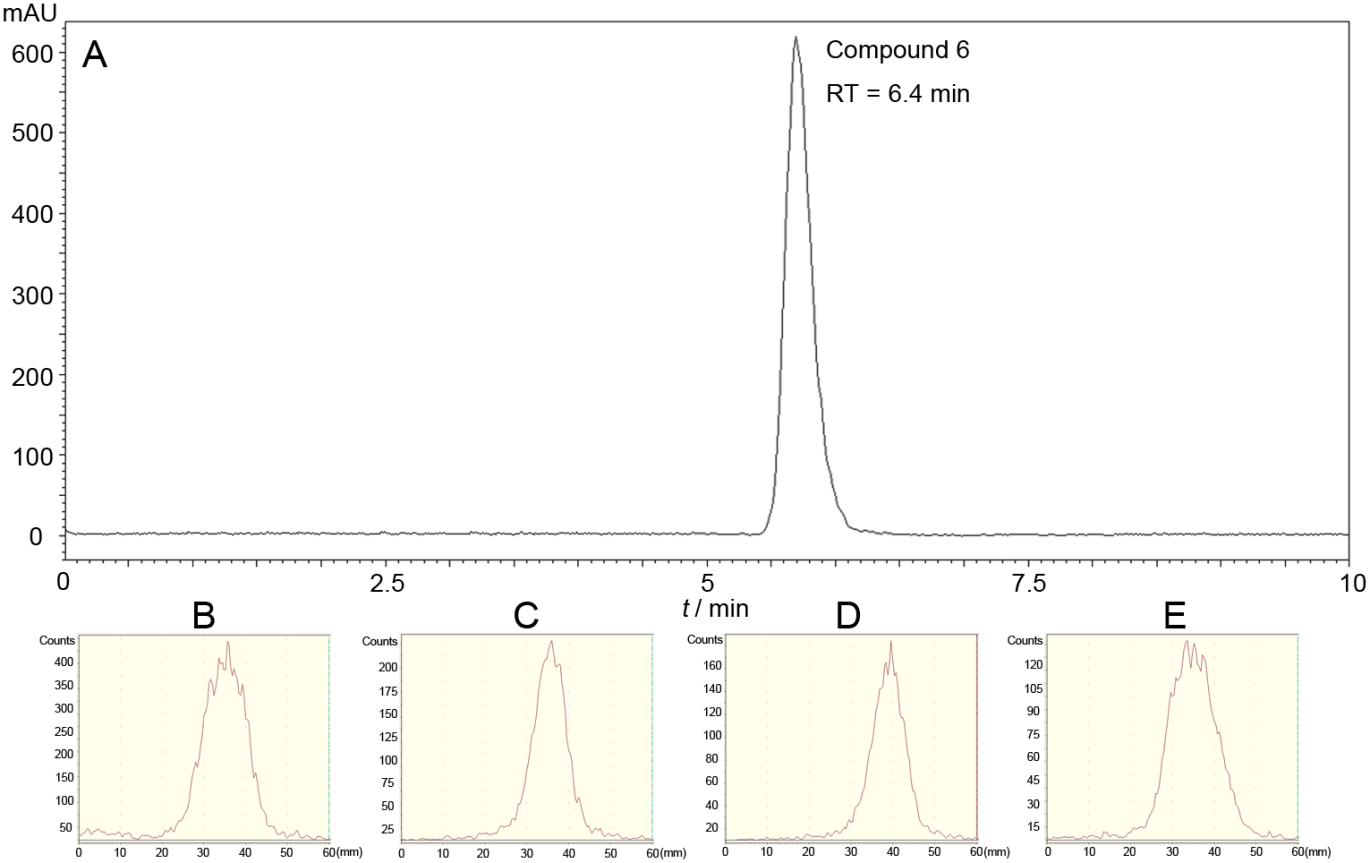
**(A)** HPLC analysis of compound 6. **(B)** Radio-TLC analysis of ^18^F-FEL saline. *In vivo* stability evaluation of ^18^F-FEL in plasma **(C)** and urine **(D)** 15 min post-injection. *In vitro* stability analysis of ^18^F-FEL in fetal bovine serum incubated at 37 °C for 120 min **(E)**.

The retention time of compound 6 was 6.4 min (Figure 1A) as measured by HPLC analysis, and the R_f_ value of ^18^F-FEL was 0.65. During the automated synthesis, the dilution was passed through three cartridges, after 5 min of base-catalyzed hydrolysis. IC-H cartridge was used for the neutralization of NaOH and absorption of cation (as K_222_/K^+^ complex). Excess ^18^F ions that were not combined were absorbed by the Alumina B cartridge. In addition, the polymers (carbohydrates yield under high temperature, usually with dark color) and partly reacted intermediates (low polarity compounds with one or more acetyl group) were trapped on the C18 cartridge. Radiosynthesis of ^18^F-FEL was completed in 30 min with a yield of 20 ± 5% (*n* = 10, non-decay-corrected). The specific activity was greater than 14.2 ± 7.1 GBq/*μ*mol (*n* = 10). The radiochemical purity was greater than 99% as analyzed by radio-TLC (Figure 1B).

### Pharmacological assays

Following the *in vivo* stability test in plasma and urine, 15 min after intravenous injection, the percentage of intact probe was measured to be no less than 94% (Figure 1C and 1D). Almost no dehalogenation was detected and this was further corroborated by a low radiotracer uptake in bone, using a biodistribution study (Table 1). In the *in vitro* stability assay, ^18^F-FEL in fetal bovine serum was greater than 95%, even at the longer time point of 120 min (Figure 1E).

**Table 1:** Biodistribution of ^18^F-FEL in T3M4 tumor-bearing mice after injection

Organ	Time after injection (min)			
	5	30	60	120
Blood	9.58 ± 0.51	2.31 ± 0.67	0.87 ± 0.31	0.29 ± 0.12
Brain	0.67 ± 0.23	0.16 ± 0.04	0.10 ± 0.02	0.06 ± 0.03
Heart	3.81 ± 0.62	0.88 ± 0.25	0.38 ± 0.10	0.20 ± 0.04
Lung	2.29 ± 0.35	1.91 ± 0.42	0.71 ± 0.37	0.38 ± 0.11
Liver	2.12 ± 0.21	1.36 ± 0.07	0.83 ± 0.23	0.59 ± 0.09
Pancreas	2.90 ± 1.28	0.69 ± 0.33	0.63 ± 0.34	0.29 ± 0.07
Kidney	19.4 ± 4.76	9.59 ± 1.80	4.35 ± 1.53	2.08 ± 0.74
Spleen	1.89 ± 0.62	0.77 ± 0.19	0.56 ± 0.30	0.30 ± 0.17
Intestine	2.57 ± 0.30	0.94 ± 0.16	0.56 ± 0.35	0.20 ± 0.07
Muscle	2.50 ± 0.15	0.91 ± 0.12	0.77 ± 0.30	0.15 ± 0.07
Stomach	3.60 ± 0.64	1.03 ± 0.33	0.82 ± 0.61	0.24 ± 0.17
Bone	1.34 ± 0.23	0.60 ± 0.26	0.31 ± 0.02	0.12 ± 0.02
Tumor	6.67 ± 0.42	3.24 ± 0.11	1.03 ± 0.09	0.65 ± 0.12

Note: Data are mean %ID/g ± SD (*n* = 5).

**Figure 2:**
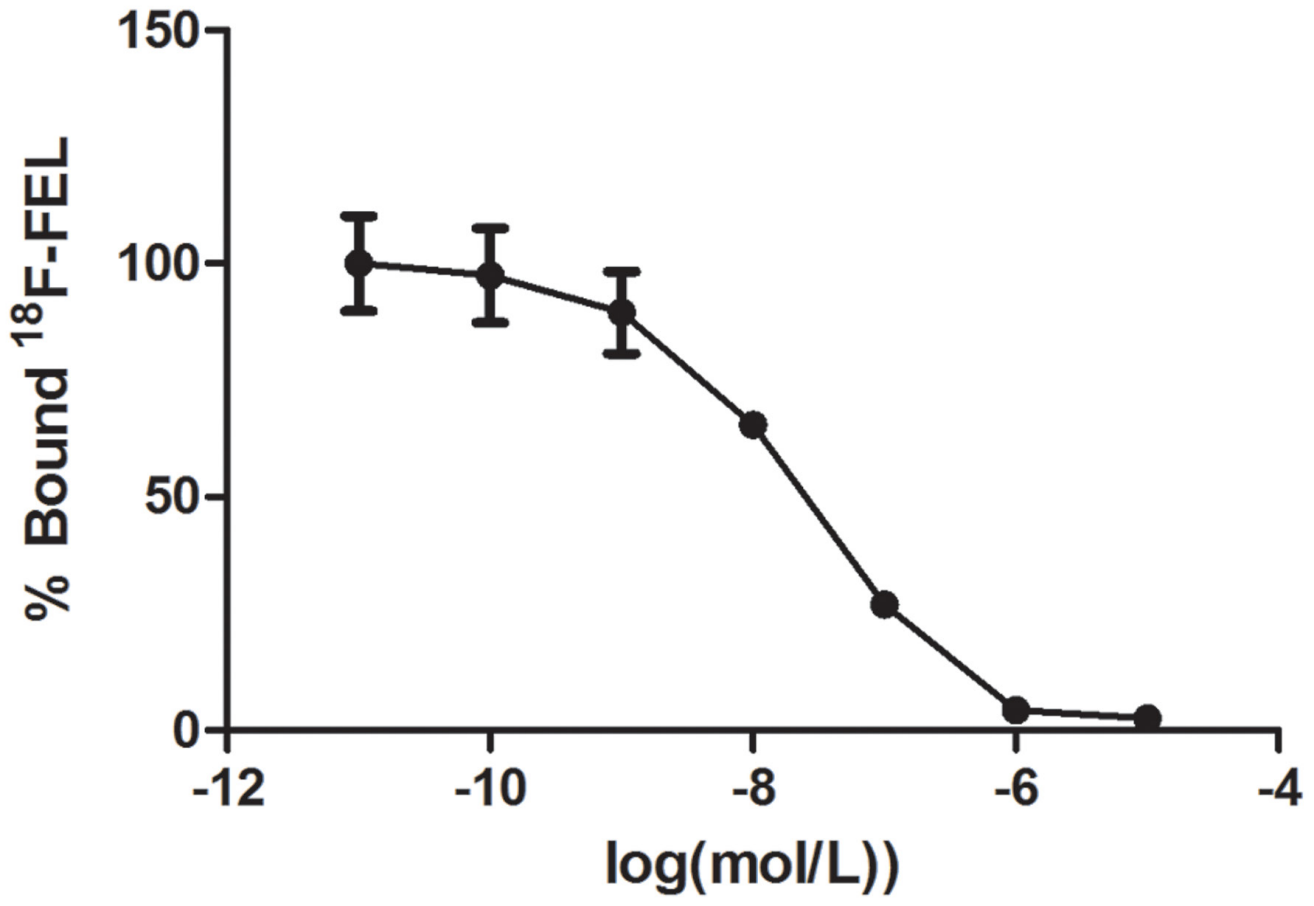
Inhibition of ^18^F-FEL binding to human HIP/PAP protein immobilized on PVC plates by *β*-D-lactose The experiment was carried out in duplicate and repeated 3 times.

The IC50 data obtained for ^18^F-FEL for binding to human HIP/PAP coated on PVC plates was calculated to be 23.0 ± 4.0 nM (Figure 2). The graph was plotted using GraphPad Software.

With a log *P* of −2.62 ± 0.12, ^18^F-FEL was strongly hydrophilic, a characteristic which also explains the predominant accumulation in kidney (renal system) and low uptake in liver (hepatic system) in micro-PET images and biodistribution studies.

### Biodistribution studies

^18^F-FELwas efficiently and rapidly cleared from blood via the renal system, although with low retention in the kidney (Table 1). Tumors had a high uptake of radioactivity at 5 min (6.67 ± 0.42 %ID/g) post-injection and declined gradually. Accumulation of the radiotracer in the liver and spleen was low, with absolute values of 2.12 ± 0.21 and 1.89 ± 0.62 at 5 min post-injection, which decreased to 0.59 ± 0.09 and 0.30 ± 0.17 %ID/g rapidly at 120 min, respectively. The low radioactivity uptake levels measured in bone were an indicator that defluorination *in vivo* did not occur.

### Small animal PET imaging

**Figure 3:**
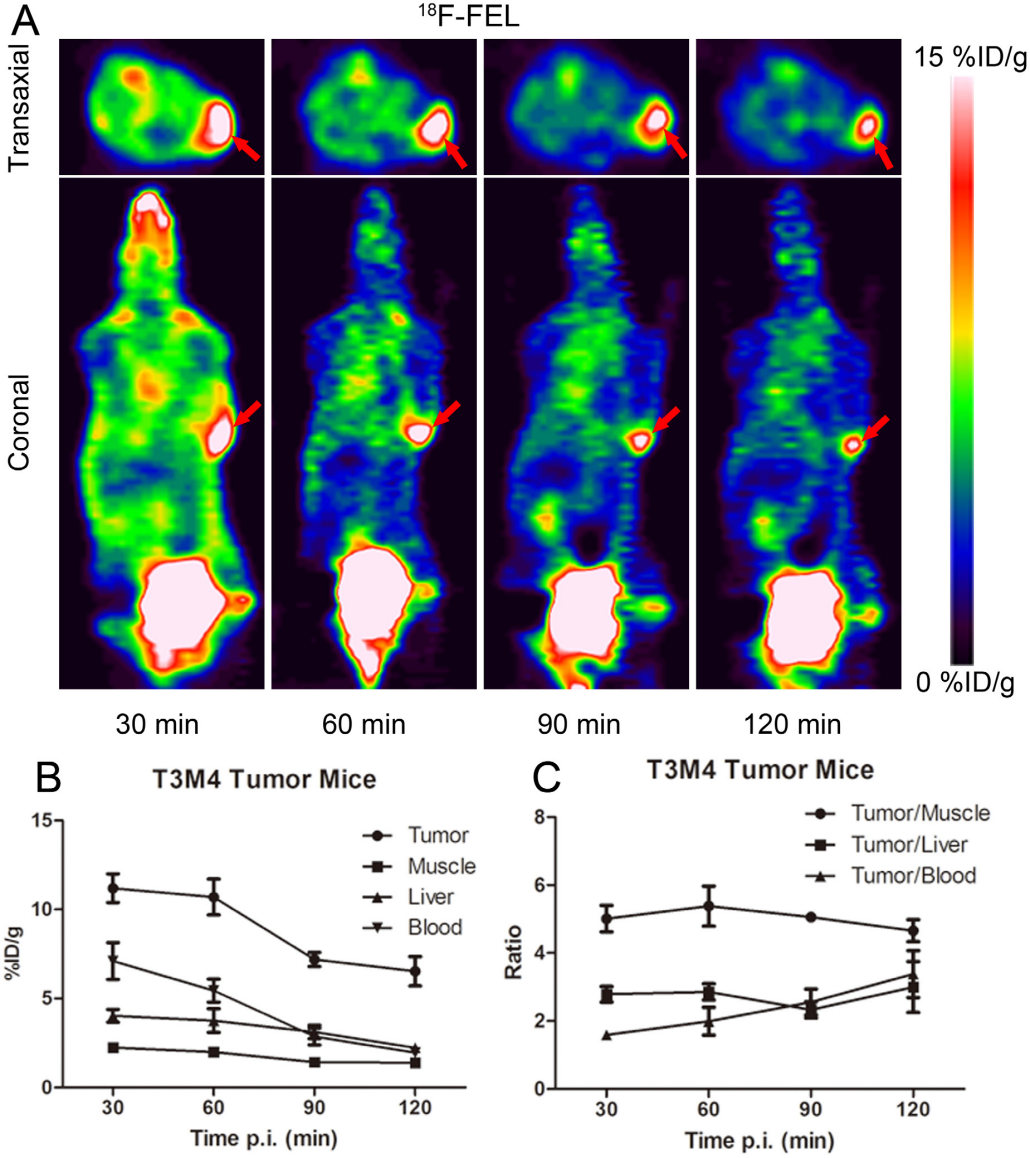
**(A)** Coronal and transaxial *in vivo* PET imaging obtained 30, 60, 90 and 120 min after injection of ^18^F-FEL in a T3M4 tumor-bearing model. Time-activity curves showing the ^18^F-FEL-derived radioactivity concentration in tumor and in different organs **(B)** and tumor-to-organ ratios **(C)**. Note: Data are mean %ID/g ± SD (*n* = 4). (Tumors are marked by red arrows).

**Figure 4:**
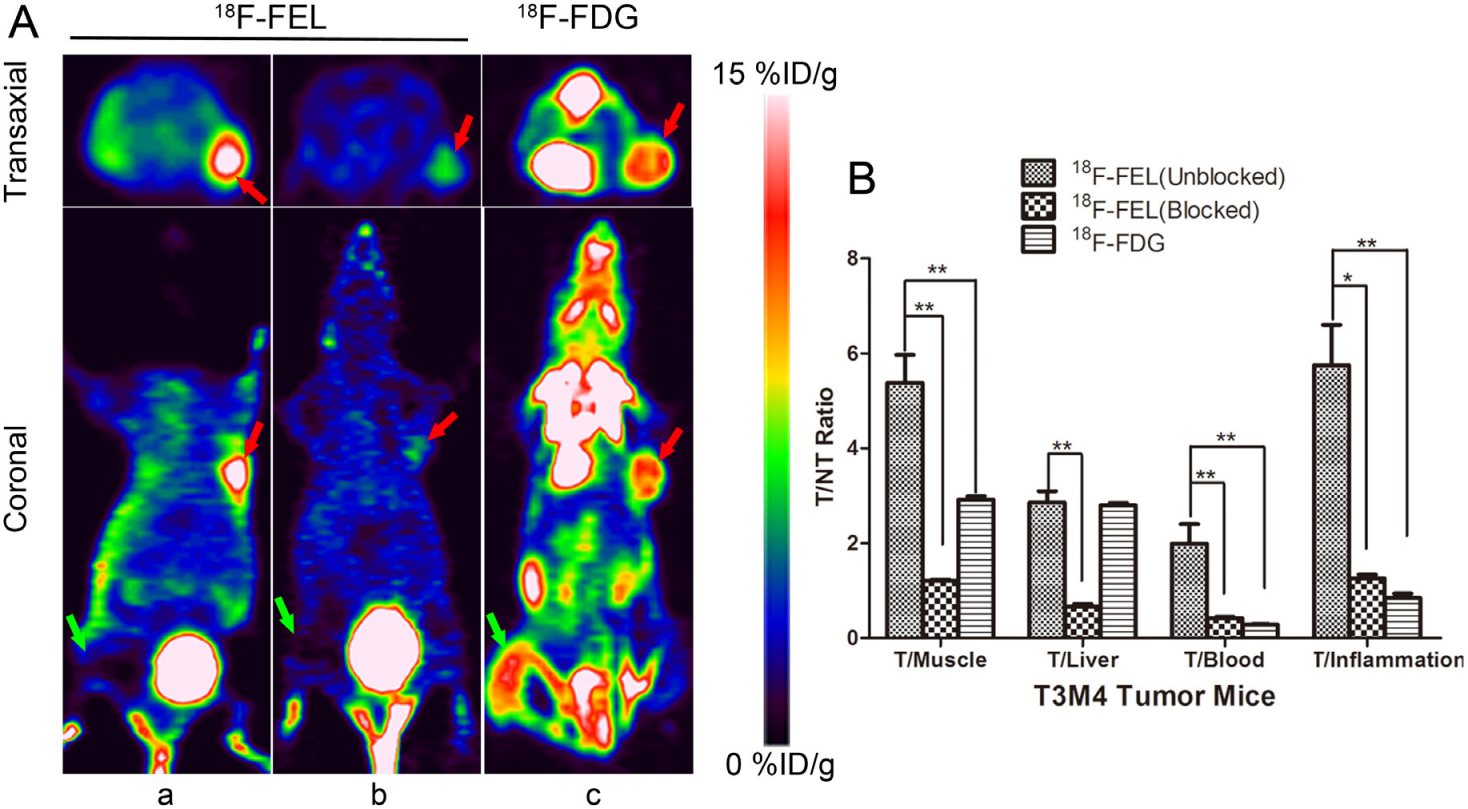
**(A)** PET imaging of T3M4 tumor-bearing inflammation nude mice 60 min after injection of ^18^F-FEL (a) and ^18^F-FDG (c). PET images 60 min after co-injection of *β*-D-lactose (15 mg/kg) (b). **(B)** The uptake ratio of: tumor-to-organ were summarized. Note: * = *P* < 0.05, ** = *P* < 0.01. (Tumors are marked by red arrows and inflammatory lesions are marked by green arrows).

**Figure 5:**
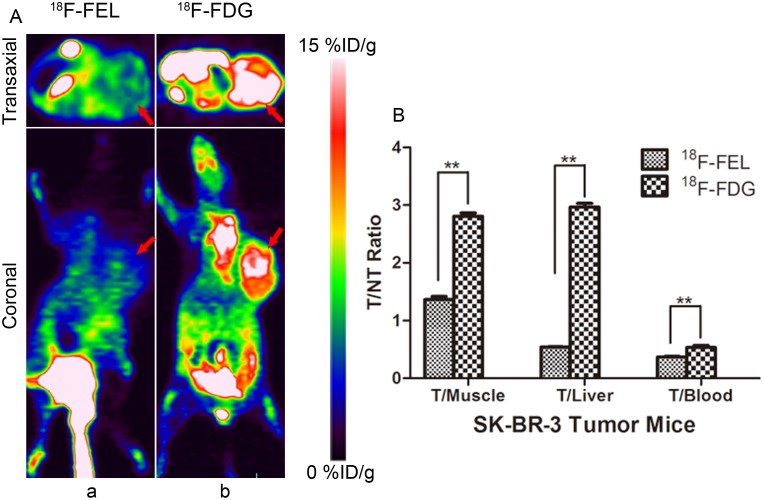
**(A)** PET imaging of SK-BR-3 xenografts 60 min after injection of ^18^F-FEL (a) and ^18^F-FDG (b) (n = 3). **(B)** The uptake ratio of: tumor-to-organ were calculated. Note: * = *P* < 0.05, ** = *P* < 0.01. (Tumors are marked by red arrows).

Representative PET images obtained in T3M4 (Figure 3A and 4A) and SK-BR-3 (Figure 5A) mice after injection of ^18^F-FEL and ^18^F-FDG. ^18^F-FEL was accumulated in T3M4 tumor 30 min post-injection, and declined slowly over 120 min (Figure 3A). The specific uptake value of tumor, muscle, liver and blood and the uptake ratio of target to non-target (T/NT) were summarized in Figure 3B and 3C. Predominant urinary excretion of the tracer was represented by the high focal activity concentration in the bladder at all time points.

In T3M4-inflammation mice, tumors were clearly delineated 60 min after injection of ^18^F-FEL, with low background and non-accumulation in inflammatory tissue (Figure 4a). However, in the same batch of mice, ^18^F-FDG uptake was in organs such as tumor, brain, heart, brown adipose tissue and inflammatory tissue (Figure 4c).

To visualize the extent of HIP/PAP-specific T3M4 tumor uptake of ^18^F-FEL, micro-PET competition studies using *β*-D-lactose as competitors were also performed. A dramatic decrease in accumulation of radiotracer was observed in tumors, which indicated a high affinity of ^18^F-FEL to HIP/PAP (Figure 4a and 4b). The T/NT ratio in T3M4 and SK-BR-3 tumor-bearing mice after injection of ^18^F-FEL and ^18^F-FDG are summarized in Figure 4B.

In order to visualize the selectivity of ^18^F-FEL to HIP/PAP, SK-BR-3 breast tumor-bearing mice with no HIP/PAP expression (which was confirmed by IHC and western blot), were imaged using ^18^F-FEL and ^18^F-FDG, respectively. As expected, the radioactivity uptake in SK-BR-3 tumor was low for ^18^F-FEL but high for ^18^F-FDG (Figure 5a and 5b).

### Immunostaining and western blot

**Figure 6:**
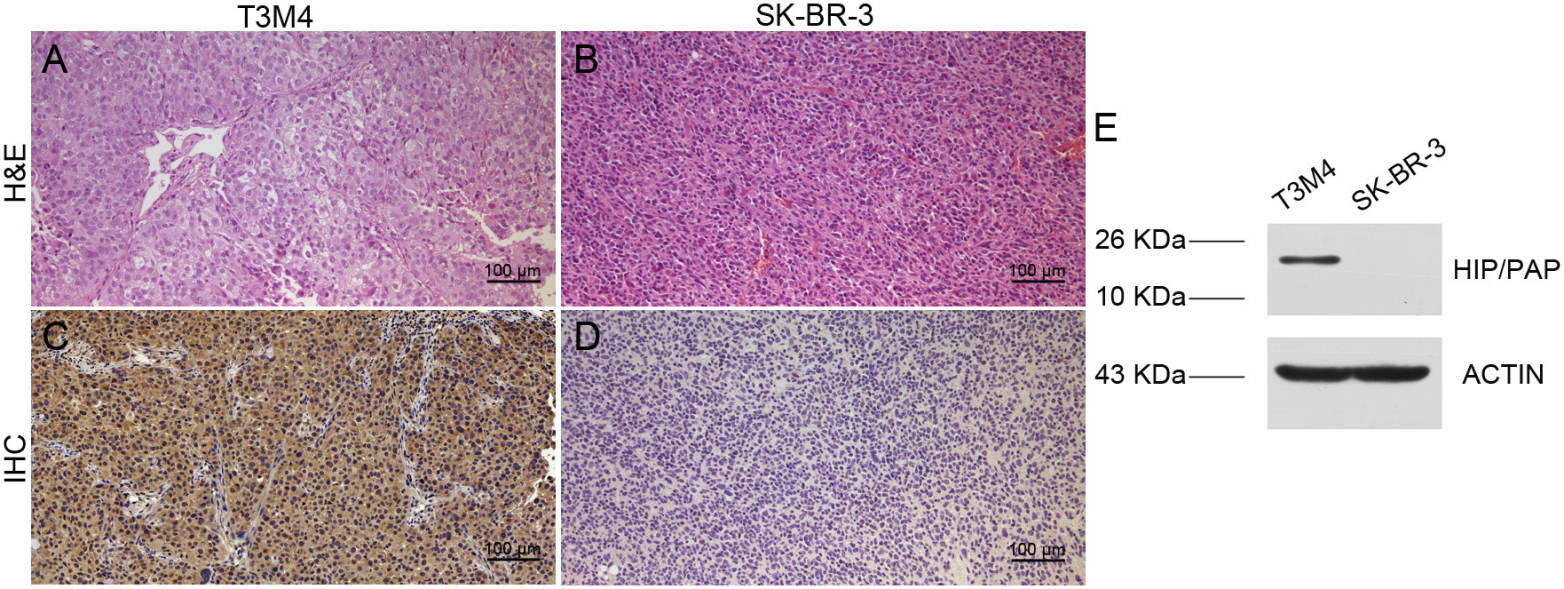
H&E **(A&B)** staining and IHC **(C&D)** of HIP/PAP in T3M4 and SK-BR-3 tumor tissues. Western blot analysis of HIP/PAP expression in T3M4 and SK-BR-3 tumor cells **(E)**.

Paraffin wax embedded sections from T3M4/SK-BR-3 tumors, dissected from a mouse that had previously undergone micro-PET, were cut and stained for H&E and immunostaining of HIP/PAP. The tumor section slices were incubated with anti-HIP/PAP antibody. Positive staining was found in the plasma of T3M4 tumors, but the staining of SK-BR-3 tumors was found to be negative (Figure 6). This finding was further corroborated by western blot, which indicated overexpression of HIP/PAP protein in T3M4 cells, but no expression in SK-BR-3 cells.

## DISCUSSION

In this study we described the synthesis of a novel precursor 5 and a fully automated preparation of ^18^F-FEL in 30 minutes, which was significantly shorter than 90 minutes - the shortest time in previously reported papers [18–20]. The radiosynthesis yield of 20 ± 5% is lower than that of the nosylate-FEL precursor (the yield is 65%), similar to mesylate-FEL precursors (the yield is 21%), but still higher than those obtained from bromo- and tosylate precursors, 9% and 11%, respectively [20]. In the hydrolysis step, we abolished the MeOH/NaOMe system and chose the NaOH/EtOH system instead, to avoid potential toxicity of MeOH, and in order to make it safer for clinical use.

The precursor synthesis was completed in three steps with a total yield of 18%. However, the triflate-compound 5 was not completely stable at room temperature and needed to be stored at -20°C or below. It was also essential to purify the precursor using flash chromatography after every two months before use.

In the radiolabeling procedure, the retention time of compound 6 was similar to the value previously reported [13, 19, 20], whereas the slightly different R_f_ values of ^18^F-FEL might be due to the different types of TLC we used.

^18^F-FDG is the most widely used tracer for imaging abnormal glucose uptake related disease and could be metabolized *in vivo* [21–25]. Unlike ^18^F-FDG,^18^F-FEL showed good stability both *in vitro* and *in vivo* (Figure 1B-1E). PET images and biodistribution studies indicated that ^18^F-FEL *in vivo* had reasonable stability, as no defluorination was observed (Figure 3-5). Our findings were identical to the previously reported biodistribution study [18].

The *in vivo* pharmacokinetics of ^18^F-FEL were characterized by fast radiotracer distribution, clearance from non-target tissues and rapid accumulation in the tumor xenografts (Table 1). ^18^F-FEL, a hydrophilic molecule, showed almost exclusive renal excretion and low accumulation in the hepatobiliary system which was in accordance with previous findings [18]. Low retention in kidney was also observed and it did not readily cross the blood-brain barrier, as evidenced by no uptake in the brain. No dehalogenation of ^18^F-FEL was observed *in vivo*, as evidenced by low levels of radiotracers in bone until the end of the experiment.

The specificity of ^18^F-FEL to mouse HIP/PAP was confirmed by *in vitro* autoradiography and IHC (orthotopic L3.6pl-GL^+^ pancreatic xenografts) using *β*-D-lactose as a blocking reagent [3, 18]. Based on these findings, we further performed the blocking experiments with PET study. Co-injection of a blocking dose of *β*-D-lactose led to the expected reduction in tumor uptake (Figure 4a and 4b). The tumor uptake of ^18^F-FEL declined from 10.7 ± 1.01 to 2.36 ± 0.11 %ID/g at 60 min post-injection, and the ratio of tumor-to-muscle, tumor-to-blood and tumor-to-liver decreased from 5.38 ± 0.59 to 1.20 ± 0.03, from 1.99 ± 0.41 to 0.42 ± 0.03 and from 2.86 ± 0.24 to 0.67 ± 0.05, respectively (Figure 4B, *P* < 0.01). The ratio of tumor-to-inflammation declined from 5.75 ± 0.85 to 1.26 ± 0.08 (*P* < 0.05). The comparison of T/NT ratios obtained under blocking conditions revealed a high affinity of ^18^F-FEL to HIP/PAP.

^18^F-FEL distributed through the whole body with higher T/NT ratio except tumor-to-liver, compared to ^18^F-FDG. In the PET study of T3M4 xenografts, the tumor-to-muscle, tumor-to-blood and tumor-to-inflammation ratios found for ^18^F-FDG at 60 min post-injection (2.95 ± 0.15, 0.28 ± 0.02 and 0.85 ± 0.09) were substantially lower than those found for ^18^F-FEL (Figure 4B, *P* < 0.01). However, no obvious difference could be observed in the tumor-to-liver ratio between ^18^F-FEL (2.87 ± 0.24) and ^18^F-FDG (2.83 ± 0.10, *P* > 0.05).

For the evaluation of specific binding ability of ^18^F-FEL to HIP/PAP, SK-BR-3 xenografts were selected for PET imaging as comparison (Figure 5A). No accumulation of radioactivity in the tumor region could be detected at 60 min post-injection, and the uptake ratio of tumor-to-muscle, tumor-to-liver and tumor-to-blood were 1.37 ± 0.05, 0.54 ± 0.01 and 0.37 ± 0.01. In comparison, the ratios increased to 2.81 ± 0.06, 2.97 ± 0.06 and 0.54 ± 0.03 respectively, when imaged with ^18^F-FDG (Figure 5B, *P* < 0.01).

To prove the positive expression of HIP/PAP in T3M4 cell lines, we performed *in vitro* experiments. Data from *in vitro* IHC analysis demonstrated exclusive HIP/PAP localization in the plasma of T3M4 pancreatic tumor cells (Figure 6C). Western blot experiments further confirmed the overexpression of the HIP/PAP protein (Figure 6E).

However, HIP/PAP is also overexpressed in pancreatic acinar cells compared to normal pancreas in pancreatitis [14, 15, 19]. Whether ^18^F-FEL could differentiates mouse pancreatic cancer from pancreatitis should be determined with genetically engineered mouse models in further studies.

## MATERIALS AND METHODS

### General

All chemicals obtained commercially were of analytical grade (Sigma-Aldrich, USA) and used without further purification unless otherwise stated. Solid-phase extraction cartridges (Sep-Pak Light QMA, Plus Long Alumina B and Plus Short and Light C18 cartridges) were obtained from Waters (Milford, MA, USA) and IC-H Maxi-Clean cartridge was purchased from Altech (Illinois, USA). Fetal bovine serum was purchased from HyClone (Thermo Scientific, USA) and stored below -20°C before use. Millexs-GS 0.22 *μ*m filters units were purchased from Merck Millipore Ltd. Human HIP/PAP (purity > 97% as determined by SDS-PAGE) was purchased from the Sino Biological Inc (Beijing, China).

^18^F-FDG was prepared as previously reported [26]. The synthesis of ^18^F-FEL was performed on the PET-MF-2V-IT-I synthesis module (PET Co. Ltd., Beijing, China). No-carrier-added [^18^F] fluoride was produced through the nuclear reaction ^18^O (p,n) ^18^F by irradiation of more than 95% [^18^O] enriched water target with 10 MeV proton beam on the Siemens PET trace cyclotron. Radioactivity was measured by a CRC-15 PET Radioisotope Dose Calibrator (Capintec. Inc, USA). All reactions were carried out under anhydrous conditions using flamed-dried glassware with freshly distilled solvents, unless otherwise noted. Column chromatography was performed on silica gel (200-300 mesh). ^1^H NMR and ^13^C NMR spectra were recorded on an Avance III Bruker-600 MHz spectrometer at 25°C using tetramethylsilane (TMS) as an internal reference. High-resolution mass spectra (HRMS) were measured on Waters Xevo G2 Q-TOF spectrometer. Elemental analysis data were recorded on a Vario EL-III elemental analyzer.

### Preparation of 1'-trifluoromethanesulfonylethyl-2',3',6',2,3,4,6-hepa-*O*-acetyl-*β*-D-lactose 5

**Figure 7:**
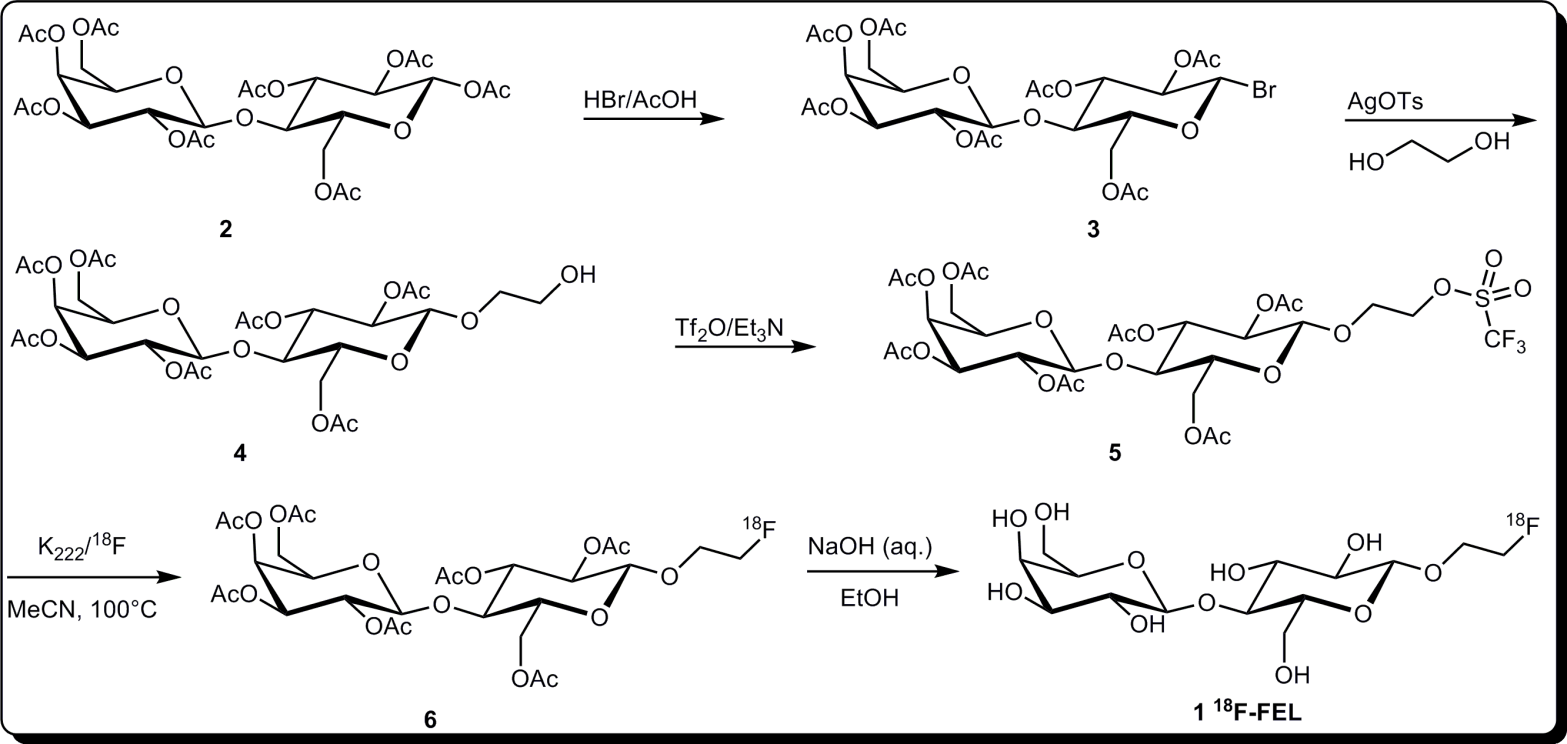
Schematic to summarize the organic synthesis of triflate-precursor 5 and radiosynthesis of ^18^F-FEL

All synthetic steps of the precursors were performed similar to the steps reported elsewhere [19, 20] except the synthesis of triflate-precursor 5 (Figure 7). Briefly, under ice bath conditions, a mixture of trifluoromethanesulfonic anhydride (Tf_2_O, 0.67 g, 3.0 mmol) dissolved in THF (10 mL) was added dropwise to a solution of 1'-hydroxyethyl-2',3',6',2,3,4,6-hepta-O-acetyl-*β*-D-lactose 4 (1.0 g, 1.5 mmol) in tetrahydrofuran (THF, 10 mL) and triethylamine (TEA, 0.5 mL, 3.6 mmol). After the addition, the reaction was warmed to room temperature and stirred overnight. The solvent was evaporated under reduced pressure and the crude product was purified by flash chromatography on a silica gel column using a mixture of ethyl acetate and petroleum ether 60-90 (1:4-1:5) to obtain precursor 5 in 80% yield as a white incompact solid. ^1^H NMR (CDCl_3_) *δ*: 5.35 (d, *J* = 3.0 Hz, 1H), 5.20 (t, *J* = 9.1 Hz, 1H), 5.11 (dd, *J* = 7.9, *J* = 10.4 Hz, 1H), 4.96 (dd, *J*=2.8Hz, *J*=10.9Hz, 1H), 4.92 (m, 1H), 4.49-4.57 (m, 3H), 4.06-4.16 (m, 5H), 3.81-3.90 (m, 4H), 3.61-3.65 (m, 1H), 2.16 (s, 3H), 2.13 (s, 3H), 2.07 (s, 3H), 2.06 (s, 3H), 2.05 (s, 6H), 1.97 (s, 3H).^13^CNMR (CDCl_3_) *δ*: 170.34, 170.13, 170.06, 169.78, 169.71, 169.64, 169.07, 101.10, 100.28, 76.01, 74.73, 72.85, 72.65, 71.26, 70.97, 70.74, 69.15, 66.62, 66.36, 61.69, 60.79, 20.78, 20.63, 20.51. HRMS (ESI): calculated for C_29_H_43_F_3_NO_21_S [M + NH_4_]^+^ 830.2000, found 830.1993.

### [^18^F]Radiofluorination conditions for ^18^F-FEL

^18^F-FEL was prepared using a two-step reaction described in Figure 7. [^18^F]fluoride (5-10 mCi) was trapped on a Light QMA cartridge and then eluted with a phase transfer catalyst solution. The eluent was then added into a 10 mL test tube and evaporated at 90°C under nitrogen bubbling. The residue was azeotropically dried with 1.5 mL anhydrous acetonitrile (CH_3_CN) at 90°C. Precursor 5 (10 mg) was dissolved in 1.0 mL anhydrous CH_3_CN. The solution was then added into the test tube containing the dried residue prepared above. The mixture was then bubbled with nitrogen and heated at 80°C for 20 min. The reaction was quenched by the addition of 10 mL of water. The mixture was loaded on a Sep Pak C18 Plus Light cartridge and the cartridge was flushed with 10 mL water. The intermediate 6 was eluted with 0.5 mL 75% ethanol and analyzed by radio-HPLC system. The HPLC was equipped with a gamma ray radiodetector and a UV detector [Waters system: Waters C18 column (4.6 × 250 mm); mobile phase: 1 mL/min with an eluent of CH_3_CN/H_2_O 55:45]. Finally, 1.0 mL 2N sodium hydroxide (NaOH) was added to the tube and heated at 80°C for 5 min. The reaction was monitored by analytical thin layer chromatography (TLC pre-coated on silica gel 60F254 pre-coated on aluminium plate) with developing solvent MeOH/H_2_0 (95:5) and radioactivity was measured on a Mini-Scan and Flow Counter (Bioscan Inc., USA).

### Automatic radiosynthesis of ^18^F-FEL

**Figure 8:**
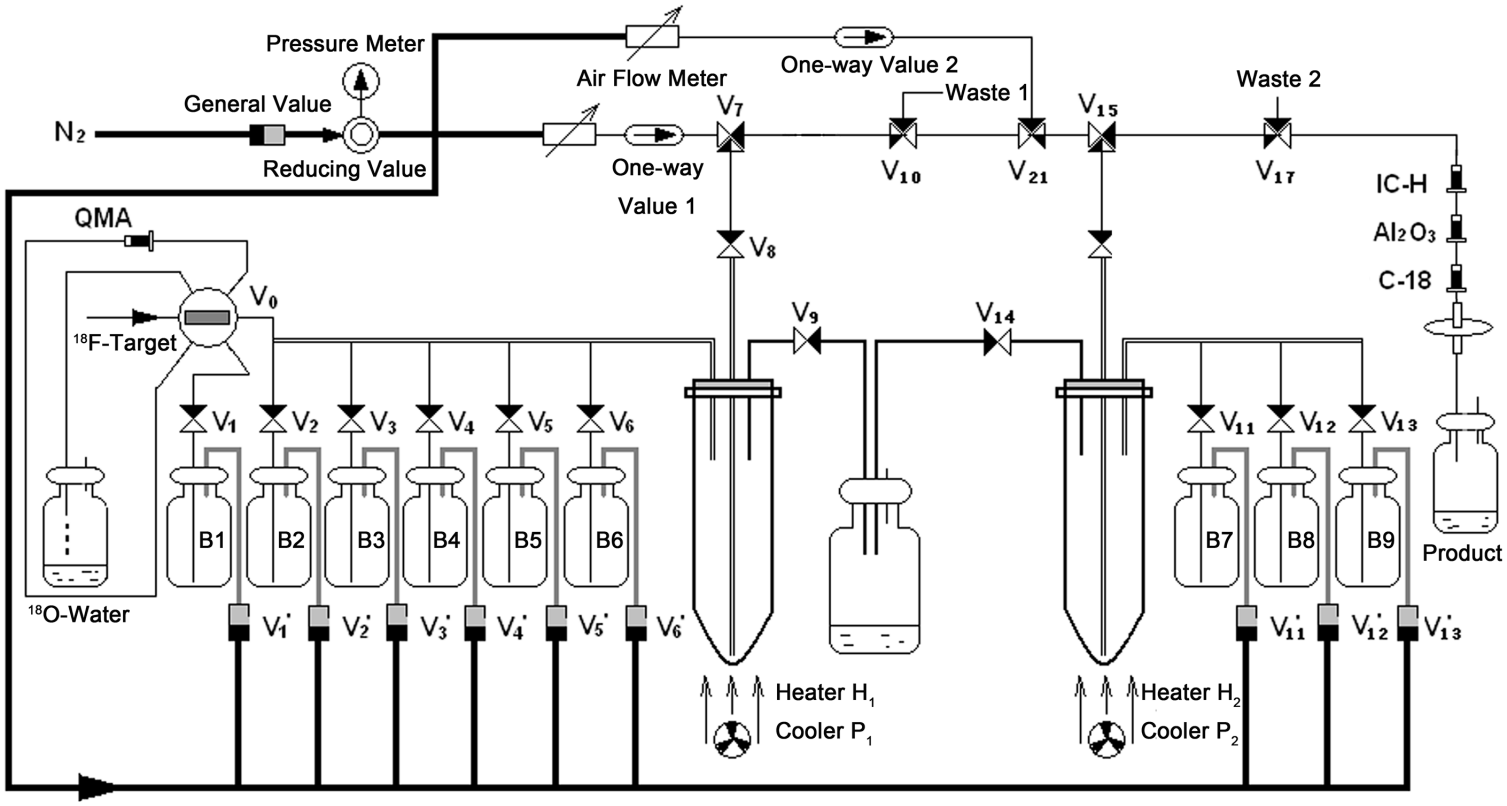
Schematic diagram of the automated synthesis of ^18^F-FEL on the PET-MF-2V-I synthesis module

The fully automated radiosynthesis of ^18^F-FEL was carried out with a PET-MF-2V-IT-I module (Figure 8). The reagents for the radiosynthesis were stored in the reagent vials. Vial B1: 1.0 mL solution containing 15 mg Kryptofix 222 (K_222_), 3 mg K_2_CO_3_, 0.9 mL CH_3_CN and 0.1 mL water; Vial B2: 1.5 mL anhydrous CH_3_CN; Vial B3: 10 mg precursor 5 dissolved in 1.0 mL anhydrous CH_3_CN; Vial B4: a mixture of 0.9 mL 2N NaOH and 0.1 mL ethanol; Vial B5: 5.0 mL water.

The PET-MF-2V-IT-I module was operated in the following process: [^18^F]fluoride (400-600 mCi) produced by a Siemens cyclotron using the ^18^O(p,n) ^18^F reaction, was trapped on a Light QMA cartridge (pre-conditioned with 8 mL 1N NaHCO_3_ and 10 mL water) and then eluted with a phase transfer catalyst solution in vial B1 to the reaction tube of the module. The eluent was evaporated at 90°C under a stream of nitrogen. Furthermore, the residue was azeotropically dried with 1.5 mL CH_3_CN (in vial B2) under the same conditions. After cooling for 60 s, the precursor solution in vial B3 was added to the reaction tube containing the dried residue prepared above and heated to 80°C for 20 min. The solution in the reaction tube was cooled for 60 s and the solvent was bubbled with nitrogen at 40°C until dry. After cooling for 60 s, the base solution in vial B4 was added to the reaction vessel and the mixture was heated at 80°C for 5 min. The reaction mixture was then quenched to room temperature, and ethanol was evaporated until dry at 40°C under reduced pressure. After 5 mL water (in vial 5) was added to the reaction tube, the dilution was passed through the three cartridges including IC-H cartridges (pre-conditioned with 10 mL water), Sep Pak Alumina B Plus Long cartridges (pre-conditioned with 10 mL water) and Sep PakC18 Plus Short cartridges (pre-conditioned with 10 mL ethanol and 10 mL water). Finally, the final product ^18^F-FEL saline was sterilized by passing through a Millipore filer (0.22 *μ*m, 25 mm) into a sterile product vial (10 mL size).

### Animal models

All animal experiments were performed in adherence with the Peking Union Medical College Hospital (PUMCH) guidelines for the use of laboratory animals and were approved by PUMCH Clinical Center Animal Care and Use Committee. The pancreatic cancer tumor-bearing models were generated by subcutaneous injection of 5 × 10^6^ human T3M4 pancreatic cancer cells (kindly supplied by Prof. Taiping Zhang from the Department of General Surgery, Peking Union Medical College Hospital) and SK-BR-3 breast cancer cells (kindly supplied by Prof. Wei Ge from Department of Immunology, Institute of Basic Medical Sciences), into the right shoulder of female athymic nude mice (Laboratory Animal Center of PUMCH). Tumor sizes were monitored with a vernier caliper. When the tumor reached approximately 3-5 mm in diameter, T3M4 tumor-bearing mice were intramuscularly injected with 0.2 mL turpentine into the left hind leg muscle [27, 28]. After 72 h, those tumor-bearing mice with a visible mass in left thigh muscle, were selected and used as models with aseptic inflammation.

### *In vivo* and *in vitro* stability

For *in vivo* stability tests, balb/c mice were injected intravenously with a dosage of 18.5 MBq (500 *μ*Ci) of ^18^F-FEL in 0.2 mL sterile saline. Urine and blood samples were collected at 15 min post-injection and blood was centrifuged (8000 rpm, 10 min) to separate out the plasma. Urine and plasma samples were analyzed by radio-TLC, as above.

For *in vitro* assays, samples of ^18^F-FEL 0.1mL (1.85 MBq, 50 *μ*Ci) dissolved in sterile saline were incubated with 0.2 mL of fetal bovine serum at 37°C with gentle shaking. An aliquot of the serum sample was analyzed using radio-TLC (MeOH/H_2_O 95:5) to determine the percentage of intact ^18^F-FEL 120 min post-injection [29, 30].

### Isolated receptor-binding assay

Competition binding experiments were performed following the procedure previously reported [31–34]. In brief, a human HIP/PAP solution (1 *μ*g/100 *μ*L) in 50 mM sodium carbonate buffer (pH 9.6) was used to coat 96-well PVC microplates which were then stored at 4°C overnight. ^18^F-FEL (3.7 kBq) was then used in a competitive binding assay with 10 *p*M-10 *μ*M *β*-D-lactose in 20 mM Tris-HCl buffer (pH 7.4, containing 150 mM NaCl, 1 mM CaCl_2_ and 1 mM MgCl_2_) for 1 h at 4°C. Wells were washed 4 times with 200 *μ*L of phosphate-buffer saline (PBS) containing 1% bovine serum albumin. Unbound radiotracer was washed out and receptor-bound activity was lysed from the plate with 2N NaOH. The radioactivity of the lysate was measured by a *γ*-counter. Three independent measurements were made, and the IC50 values were calculated by fitting the percent inhibition values using SPSS software.

### Partition coefficient determination

Octanol-water partition coefficient was determined by measuring the distribution of ^18^F-FEL in *n*-octanol and PBS (pH = 7.4). A 20 *μ*L sample of ^18^F-FEL (20 *μ*Ci) in saline was added to the Eppendorf tube containing 0.5 mL *n*-octanol and 0.5 mL PBS. After vortexing for 10 min, the tube was centrifuged (8000 rpm, 3 min) for layer separation. An aliquot of the aqueous and *n*-octanol layers was collected and measured by a *γ*-counter. Log *P* values were calculated using the following formula: log *P* = log (counts of *n*-octanol/counts of PBS, mean of *n* = 3) [34–38].

### *In vivo* biodistribution

The receptor-specific uptake was determined using adult female T3M4 tumor-bearing nude mice. ^18^F-FEL (40-80 *μ*Ci) in 0.2 mL of sterile saline was administrated (5 per group). At 5 min, 30 min, 60 min and 120 min after injection, mice were sacrificed by humane euthanasia - cervical dislocation. Blood was obtained through the eyeball, and tissues and organs of interests (brain, heart, lung, liver, pancreas, kidney, spleen, intestine, muscle, stomach, bone and tumor) were then weighed and counted. All measurements were background-subtracted and decay-corrected to the time of killing and then averaged. Radioactivity values were calculated and presented as the percentage injected dose per gram (%ID/g) of tissue.

### Small-animal PET imaging

*In vivo* PET imaging was performed using a small-animal PET scanner Inveon (Siemens Medical systems, USA). Mice bearing T3M4/SK-BR-3 xenografts were imaged by PET using ^18^F-FEL or ^18^F-FDG. In a HIP/PAP-blocking experiment, *β*-D-lactose (15 mg/kg) was co-injected with ^18^F-FEL saline into T3M4 tumor-bearing mice via the tail vein. As to the typical imaging process, intravenous injection of 0.2 mL ^18^F-FEL/^18^F-FDG saline (100-200 *μ*Ci) was followed by a 10 min static PET scan. Mice were anesthetized with isoflurane and placed on a heated pad (to provide the animal with warmth throughout the scanning). PET images were acquired at 30, 60, 90 and 120 min post-injection. After the acquisition, region of interests (ROIs), including tumor, muscle, brain and kidney were drawn over major organs on decay-corrected whole-body coronal images using Inveon Research Workplace 4.1 software. Radioactivity concentration of organs was obtained from the mean pixel values within the multiple ROI volume, and converted into MBq/mL using a conversion factor. Assuming the density of tissue was 1 g/cm^3^, the ROIs were converted to MBq/g and then divided by the administered activity, to obtain an imaging ROI-derived %ID/g.

### Immunostaining and western blot

Tumor status was further confirmed using H&E and IHC. All the tissue-sampling procedures were performed according to the corresponding protocols. Briefly, paraffin embedded tumor tissue was sectioned at 5 *μ*m and laid over glass slides, then stained by anti-HIP/PAP antibody (1:500, ThermoFisher Scientific Inc., PA5-23341), followed by secondary antibody (DAKO). The embedded tumor tissues were imaged at 200× using a Leica DM5000 microscope. Western blot was performed on the T3M4 and SK-BR-3 tumor cells to detect HIP/PAP expression [10, 39]. Briefly, 50 *μ*g of protein lysate was separated by SDS-PAGE and transferred to a PVDF membrane. The membranes were incubated with monoclonal antibodies against REG3A (1:1000) and *β*-actin (1:1000, Wuhan Goodbio technology CO., LTD), and were detected using an enhanced ECL system (Wuhan Goodbio technology CO., LTD).

### Statistical analysis

Quantitative data were expressed as mean ± SD. Statistical analysis was performed using the Student's *t*-test (SPSS 19.0). Statistical significance was defined as *P* < 0.05 (*) and *P* < 0.001 (**).

## CONCLUSION

In conclusion, we successfully established a convenient, automated synthesis method of ^18^F-FEL, assessed its potential application in PET imaging of HIP/PAP *in vivo* and found its advantage over ^18^F-FDG in differentiating tumor from aseptic inflammation in animal models.
